# 5-Meth­oxy-2,2-dimethyl-6-[(2*E*)-2-methyl­but-2-eno­yl]-10-phenyl-2*H*,8*H*-pyrano[2,3-*f*]chromen-8-one (calophyllolide)

**DOI:** 10.1107/S1600536810013577

**Published:** 2010-04-17

**Authors:** L. Kalyanaraman, R. Mohan Kumar, Peddy Vishweshwar, R. Pichai, S. Narasimhan

**Affiliations:** a Department of Chemistry, Presidency College, Chennai 600 005, India; b Asthagiri Herbal Research Foundation, 14/1, II Main road, Jayanagar, Tambaram Sanatorium, Chennai 600 047, India; c Department of Analytical Research, Discovery Research, Dr Reddy’s Laboratories Ltd, Miyapur, Hyderabad 500 049, India

## Abstract

The title compound, C_26_H_24_O_5_, was isolated from *calophyllum inophyllum* seeds. In the mol­ecule, the phenyl and 2-methyl­but-2-enoyl groups are almost orthogonal to the chromene fragment [C—C—C—C torsion angles = 81.4 (3) and −90.1 (2)°, respectively]. In the crystal packing, centrosymmetrically related mol­ecules are linked by C—H⋯O contacts into dimers, which are connected *via* further C—H⋯O inter­actions into a double chain along [010].

## Related literature

For information on weak hydrogen bonds, see: Desiraju & Steiner (1999 ▶). For the Chebychev polynomial used in the weighting scheme, see: Carruthers & Watkin, (1979 ▶).
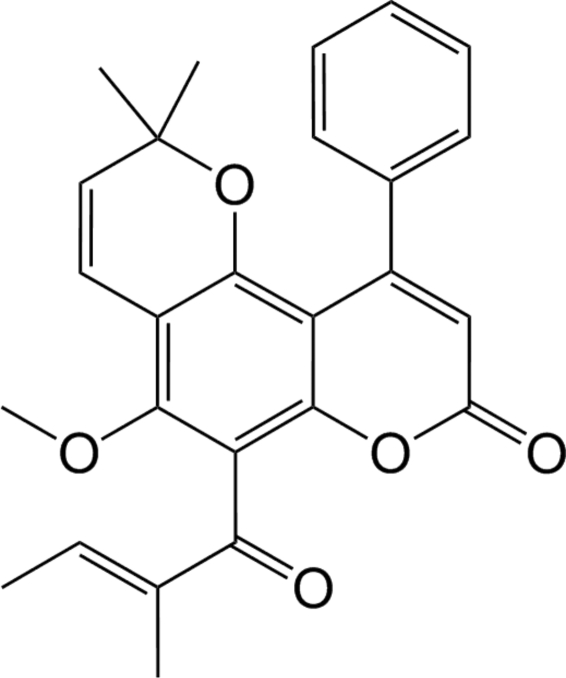


## Experimental

### 

#### Crystal data

C_26_H_24_O_5_
*M* *_r_* = 416.47
Triclinic, 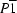

*a* = 8.943 (3) Å
*b* = 11.559 (4) Å
*c* = 12.171 (3) Å
α = 96.238 (8)°
β = 104.041 (5)°
γ = 110.301 (8)°
*V* = 1118.7 (6) Å^3^
*Z* = 2
Mo *K*α radiation
μ = 0.09 mm^−1^
*T* = 298 K
0.50 × 0.50 × 0.50 mm

#### Data collection

Rigaku Mercury diffractometer
12333 measured reflections
4480 independent reflections
2706 reflections with *F* ^2^ > 2σ(*F* ^2^)
*R*_int_ = 0.055

#### Refinement

*R*[*F* ^2^ > 2σ(*F* ^2^)] = 0.078
*wR*(*F* ^2^) = 0.092
*S* = 0.96
4480 reflections
304 parameters
H-atom parameters constrained
Δρ_max_ = 0.34 e Å^−3^
Δρ_min_ = −0.31 e Å^−3^

### 

Data collection: *CrystalClear* (Rigaku, 2005 ▶); cell refinement: *CrystalClear*; data reduction: *CrystalStructure* (Molecular Structure Corporation & Rigaku, 2006 ▶); program(s) used to solve structure: *SIR2004* (Burla *et al.*, 2005 ▶); program(s) used to refine structure: *CRYSTALS* (Betteridge *et al.*, 2003 ▶); molecular graphics: *X-SEED* (Barbour, 2001 ▶); software used to prepare material for publication: *CrystalStructure*.

## Supplementary Material

Crystal structure: contains datablocks global, I. DOI: 10.1107/S1600536810013577/tk2651sup1.cif

Structure factors: contains datablocks I. DOI: 10.1107/S1600536810013577/tk2651Isup2.hkl

Additional supplementary materials:  crystallographic information; 3D view; checkCIF report

## Figures and Tables

**Table 1:** Hydrogen-bond geometry (Å, °)

*D*—H⋯*A*	*D*—H	H⋯*A*	*D*⋯*A*	*D*—H⋯*A*
C11—H11⋯O2^i^	0.95	2.44	3.381 (3)	172
C26—H26⋯O5^ii^	0.95	2.40	3.225 (3)	145

Symmetry codes: (i) 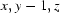; (ii) 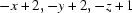.

**Fig. 1.**
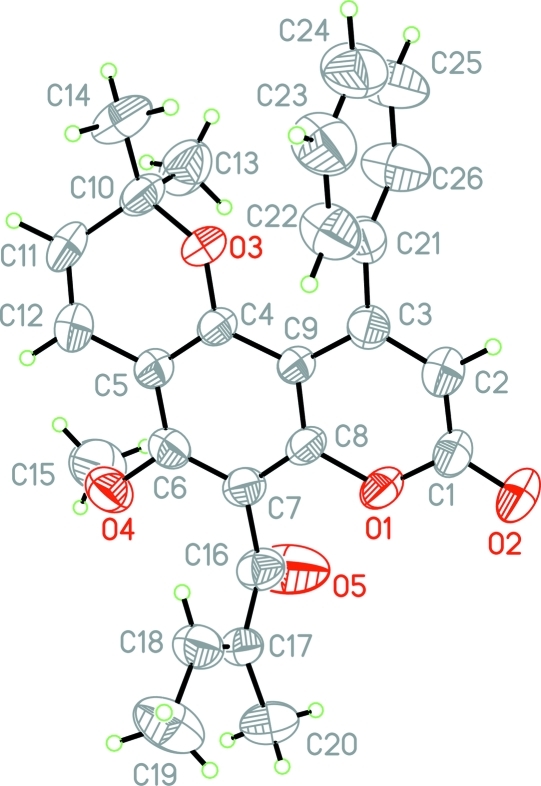
Molecular structure of (I), showing the atom numbering scheme. The displacement ellipsoids are drawn at the 50% probability level. H-atoms are shown by small circles of arbitrary radii.

**Fig. 2.**
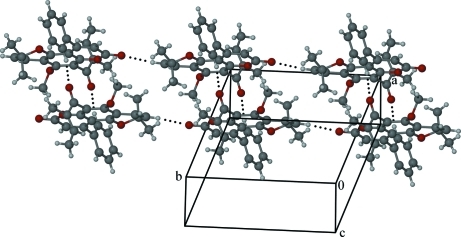
A view of the double chain in (I) sustained by C–H···O contacts shown as dashed lines..

## supplementary crystallographic information

### Comment

The molecular structure of (I), Fig. 1, shows that the phenyl and 2-methylbut-2-enoyl groups are almost orthogonal to the chromene fragment as seen in the C9–C3–C21–C22 and C6–C7–C16–C17 torsion angles of 81.4 (3)° and -90.1 (2)°, respectively. In the crystal, two molecules are linked by a pair of C26–H26···O5 contacts (Desiraju & Steiner, 1999), to form a centrosymmetric dimer, Table 1. The dimers thus formed are connected by C11–H11···O2 contacts to form a double chain along [010]; Fig. 2 and Table 1.

### Experimental

Calophyllolide (I) is the major constituent of *Calophyllum inophyllum* seed. Seeds, which were collected from the coastal Tamil Nadu (India), were dried in the shade and powdered. Soxhlet extraction was performed to extract (I) from the seed powder using *n*-hexane. The compound was purified by silica column chromatography using *n*-hexane and ethyl acetate as eluent. Compound (I) (ca. 20 mg) was dissolved in a solvent mixture comprising *n*-hexane (8 ml) and acetone (2 ml). Slow evaporation at room temperature yielded crystals after a few days.

### Refinement

The C-bound H atoms were geometrically placed (C–H = 0.95 Å) and refined as riding with *U_iso_*(H) = 1.2*U_eq_*(parent atom). A Chebychev polynomial with three parameters was used in the weighting scheme, see: Carruthers & Watkin, 1979)

### Crystal data

**Table:**

C_26_H_24_O_5_	*Z* = 2
*M**_r_* = 416.47	*F*(000) = 440.00
Triclinic, *P*1	*D*_x_ = 1.236 Mg m^−^^3^
Hall symbol: -P 1	Mo *K*α radiation, λ = 0.71070 Å
*a* = 8.943 (3) Å	Cell parameters from 4735 reflections
*b* = 11.559 (4) Å	θ = 2.5–27.4°
*c* = 12.171 (3) Å	µ = 0.09 mm^−^^1^
α = 96.238 (8)°	*T* = 298 K
β = 104.041 (5)°	Prism, colorless
γ = 110.301 (8)°	0.50 × 0.50 × 0.50 mm
*V* = 1118.7 (6) Å^3^	

### Data collection

**Table:**

Rigaku Mercury diffractometer	*R*_int_ = 0.055
Detector resolution: 7.31 pixels mm^-1^	θ_max_ = 27.4°
ω scans	*h* = −11→11
12333 measured reflections	*k* = −14→11
4480 independent reflections	*l* = −15→15
2706 reflections with *F*^2^ > 2σ(*F*^2^)	

### Refinement

**Table:**

Refinement on *F*^2^	H-atom parameters constrained
*R*[*F*^2^ > 2σ(*F*^2^)] = 0.078	Chebychev polynomial *w*ith 3 parameters (Carruthers & Watkin, 1979): 26851.8000, 36815.3000, 10053.3000
*wR*(*F*^2^) = 0.092	(Δ/σ)_max_ = 0.018
*S* = 0.96	Δρ_max_ = 0.34 e Å^−^^3^
4480 reflections	Δρ_min_ = −0.31 e Å^−^^3^
304 parameters	

### Special details

**Table:**

Geometry. Bond distances, angles etc. have been calculated using the rounded fractional coordinates. All su's are estimated from the variances of the (full) variance-covariance matrix. The cell esds are taken into account in the estimation of distances, angles and torsion angles
Refinement. Refinement was performed using all reflections. The weighted *R*-factor (wR) and goodness of fit (*S*) are based on *F*^2^. *R*-factor (gt) are based on *F*. The threshold expression of *F*^2^ > 2.0 σ(*F*^2^) is used only for calculating *R*-factor (gt).

### Fractional atomic coordinates and isotropic or equivalent isotropic displacement parameters (Å^2^)

**Table:**

	*x*	*y*	*z*	*U*_iso_*/*U*_eq_	
O1	0.76337 (17)	1.04526 (10)	0.54924 (11)	0.0565 (4)	
O2	0.6788 (2)	1.17903 (13)	0.46095 (14)	0.0901 (7)	
O3	0.61388 (16)	0.61390 (10)	0.36909 (10)	0.0549 (4)	
O4	0.97605 (19)	0.78784 (14)	0.75890 (11)	0.0674 (5)	
O5	1.1027 (2)	1.10050 (18)	0.75198 (16)	0.1159 (8)	
C1	0.6716 (2)	1.07075 (17)	0.45404 (18)	0.0615 (7)	
C2	0.5705 (2)	0.96723 (17)	0.35769 (17)	0.0598 (7)	
C3	0.5714 (2)	0.85017 (16)	0.35387 (15)	0.0478 (5)	
C4	0.7000 (2)	0.71305 (14)	0.46131 (14)	0.0454 (5)	
C5	0.7989 (2)	0.69796 (16)	0.56201 (15)	0.0508 (5)	
C6	0.8849 (2)	0.80206 (17)	0.65664 (15)	0.0519 (6)	
C7	0.8693 (2)	0.91656 (15)	0.65085 (15)	0.0484 (5)	
C8	0.7687 (2)	0.92695 (14)	0.54971 (15)	0.0449 (5)	
C9	0.6773 (2)	0.82733 (14)	0.45266 (13)	0.0417 (5)	
C10	0.6832 (2)	0.51684 (16)	0.35424 (17)	0.0589 (6)	
C11	0.7414 (3)	0.48607 (17)	0.4694 (2)	0.0740 (8)	
C12	0.8001 (3)	0.57273 (18)	0.56640 (19)	0.0688 (8)	
C13	0.8261 (3)	0.5703 (2)	0.3035 (2)	0.0845 (10)	
C14	0.5371 (3)	0.40608 (18)	0.2700 (2)	0.0821 (9)	
C15	1.1373 (3)	0.7892 (2)	0.7603 (2)	0.0913 (10)	
C16	0.9666 (2)	1.02968 (17)	0.75119 (16)	0.0569 (6)	
C17	0.8941 (2)	1.05157 (16)	0.84342 (15)	0.0540 (6)	
C18	0.7537 (2)	0.9633 (2)	0.84800 (17)	0.0637 (7)	
C19	0.6672 (3)	0.9685 (3)	0.9381 (2)	0.1058 (11)	
C20	0.9897 (3)	1.1736 (2)	0.9313 (2)	0.0908 (9)	
C21	0.4565 (2)	0.74887 (17)	0.24960 (15)	0.0530 (5)	
C22	0.3021 (2)	0.6686 (2)	0.25190 (19)	0.0747 (8)	
C23	0.1968 (3)	0.5724 (2)	0.1561 (2)	0.0879 (9)	
C24	0.2434 (3)	0.5585 (2)	0.0590 (2)	0.0906 (9)	
C25	0.3941 (3)	0.6396 (2)	0.0550 (2)	0.1014 (10)	
C26	0.5003 (3)	0.7362 (2)	0.14927 (19)	0.0842 (9)	
H2	0.50080	0.98190	0.29290	0.0750*	
H11	0.73470	0.40280	0.47280	0.0930*	
H12	0.84350	0.55350	0.63820	0.0880*	
H18	0.70430	0.88900	0.78860	0.0790*	
H22	0.26760	0.67840	0.31910	0.0850*	
H23	0.09120	0.51610	0.15790	0.0930*	
H24	0.17210	0.49190	−0.00560	0.0950*	
H25	0.42410	0.63110	−0.01430	0.1080*	
H26	0.60490	0.79210	0.14550	0.0910*	
H131	0.90770	0.53590	0.32850	0.1110*	
H132	0.87580	0.65960	0.32870	0.1110*	
H133	0.78370	0.54880	0.22140	0.1110*	
H141	0.47650	0.35420	0.31270	0.0950*	
H142	0.57630	0.35810	0.22430	0.0940*	
H143	0.46610	0.43660	0.22100	0.0950*	
H151	1.12660	0.70530	0.73490	0.1170*	
H152	1.21370	0.82260	0.83630	0.1170*	
H153	1.17770	0.83980	0.70910	0.1170*	
H191	0.55050	0.92150	0.90490	0.1330*	
H192	0.70940	0.93440	1.00040	0.1340*	
H193	0.68690	1.05410	0.96610	0.1340*	
H201	0.91510	1.20110	0.95980	0.1020*	
H202	1.06610	1.16130	0.99370	0.1020*	
H203	1.04970	1.23560	0.89570	0.1020*	

### Atomic displacement parameters (Å^2^)

**Table:**

	*U*^11^	*U*^22^	*U*^33^	*U*^12^	*U*^13^	*U*^23^
O1	0.0749 (8)	0.0356 (5)	0.0624 (7)	0.0231 (5)	0.0257 (6)	0.0064 (5)
O2	0.1485 (16)	0.0520 (8)	0.0941 (12)	0.0577 (9)	0.0467 (11)	0.0257 (7)
O3	0.0728 (8)	0.0364 (6)	0.0526 (7)	0.0220 (5)	0.0166 (6)	0.0006 (5)
O4	0.0794 (9)	0.0817 (9)	0.0477 (7)	0.0397 (8)	0.0165 (6)	0.0176 (6)
O5	0.0764 (11)	0.1121 (14)	0.1056 (14)	−0.0198 (10)	0.0414 (10)	−0.0323 (11)
C1	0.0890 (14)	0.0468 (10)	0.0662 (12)	0.0374 (10)	0.0333 (11)	0.0212 (9)
C2	0.0729 (13)	0.0520 (10)	0.0637 (12)	0.0328 (9)	0.0215 (10)	0.0180 (9)
C3	0.0525 (9)	0.0482 (9)	0.0495 (9)	0.0226 (8)	0.0214 (7)	0.0137 (7)
C4	0.0584 (10)	0.0343 (7)	0.0465 (9)	0.0180 (7)	0.0216 (7)	0.0068 (6)
C5	0.0688 (11)	0.0441 (8)	0.0489 (9)	0.0269 (8)	0.0256 (8)	0.0124 (7)
C6	0.0617 (11)	0.0575 (10)	0.0430 (9)	0.0266 (8)	0.0207 (8)	0.0140 (7)
C7	0.0533 (10)	0.0438 (8)	0.0486 (9)	0.0175 (7)	0.0196 (7)	0.0060 (7)
C8	0.0539 (9)	0.0328 (7)	0.0514 (9)	0.0179 (7)	0.0214 (8)	0.0055 (6)
C9	0.0520 (9)	0.0362 (7)	0.0413 (8)	0.0194 (7)	0.0183 (7)	0.0086 (6)
C10	0.0787 (13)	0.0371 (8)	0.0656 (12)	0.0253 (8)	0.0287 (10)	0.0042 (7)
C11	0.1114 (18)	0.0362 (9)	0.0839 (15)	0.0360 (11)	0.0336 (13)	0.0157 (9)
C12	0.1012 (16)	0.0529 (10)	0.0658 (13)	0.0406 (11)	0.0283 (12)	0.0234 (9)
C13	0.1012 (18)	0.0680 (13)	0.1079 (19)	0.0420 (13)	0.0576 (16)	0.0221 (12)
C14	0.0934 (17)	0.0468 (11)	0.0952 (17)	0.0213 (11)	0.0294 (13)	−0.0107 (10)
C15	0.0966 (18)	0.119 (2)	0.0764 (16)	0.0670 (17)	0.0208 (13)	0.0192 (14)
C16	0.0543 (11)	0.0483 (9)	0.0573 (11)	0.0117 (8)	0.0144 (8)	0.0015 (8)
C17	0.0714 (12)	0.0495 (9)	0.0412 (9)	0.0298 (9)	0.0105 (8)	0.0034 (7)
C18	0.0734 (13)	0.0721 (12)	0.0526 (11)	0.0333 (11)	0.0244 (9)	0.0116 (9)
C19	0.114 (2)	0.148 (2)	0.0715 (16)	0.055 (2)	0.0482 (16)	0.0263 (16)
C20	0.122 (2)	0.0685 (14)	0.0641 (14)	0.0308 (14)	0.0172 (13)	−0.0123 (11)
C21	0.0577 (10)	0.0509 (9)	0.0486 (9)	0.0192 (8)	0.0136 (8)	0.0150 (7)
C22	0.0653 (13)	0.0826 (14)	0.0635 (13)	0.0144 (11)	0.0186 (10)	0.0149 (11)
C23	0.0701 (15)	0.0864 (16)	0.0759 (15)	0.0031 (12)	0.0088 (12)	0.0147 (13)
C24	0.0828 (17)	0.0856 (17)	0.0683 (15)	0.0129 (14)	−0.0020 (13)	−0.0004 (13)
C25	0.0934 (19)	0.120 (2)	0.0559 (13)	0.0087 (16)	0.0222 (13)	−0.0095 (14)
C26	0.0747 (15)	0.0923 (17)	0.0604 (13)	0.0053 (13)	0.0248 (11)	−0.0031 (12)

### Geometric parameters (Å, °)

**Table:**

O1—C1	1.372 (2)	C21—C26	1.378 (3)
O1—C8	1.385 (2)	C22—C23	1.393 (3)
O2—C1	1.223 (2)	C23—C24	1.357 (4)
O3—C4	1.362 (2)	C24—C25	1.365 (4)
O3—C10	1.473 (2)	C25—C26	1.386 (3)
O4—C6	1.373 (2)	C2—H2	0.9500
O4—C15	1.433 (3)	C11—H11	0.9500
O5—C16	1.205 (3)	C12—H12	0.9500
C1—C2	1.433 (3)	C13—H131	0.9500
C2—C3	1.352 (3)	C13—H132	0.9500
C3—C9	1.450 (2)	C13—H133	0.9500
C3—C21	1.499 (3)	C14—H141	0.9500
C4—C5	1.394 (2)	C14—H142	0.9500
C4—C9	1.415 (2)	C14—H143	0.9500
C5—C6	1.412 (3)	C15—H151	0.9500
C5—C12	1.458 (3)	C15—H152	0.9500
C6—C7	1.386 (3)	C15—H153	0.9500
C7—C8	1.382 (3)	C18—H18	0.9500
C7—C16	1.519 (3)	C19—H191	0.9500
C8—C9	1.404 (2)	C19—H192	0.9500
C10—C11	1.498 (3)	C19—H193	0.9500
C10—C13	1.521 (3)	C20—H201	0.9500
C10—C14	1.517 (3)	C20—H202	0.9500
C11—C12	1.323 (3)	C20—H203	0.9500
C16—C17	1.466 (3)	C22—H22	0.9500
C17—C18	1.333 (3)	C23—H23	0.9500
C17—C20	1.502 (3)	C24—H24	0.9500
C18—C19	1.495 (3)	C25—H25	0.9500
C21—C22	1.380 (3)	C26—H26	0.9500
			
C1—O1—C8	121.76 (14)	C21—C26—C25	120.1 (2)
C4—O3—C10	117.74 (14)	C1—C2—H2	118.00
C6—O4—C15	115.00 (16)	C3—C2—H2	118.00
O1—C1—O2	116.84 (18)	C10—C11—H11	119.00
O1—C1—C2	117.20 (16)	C12—C11—H11	120.00
O2—C1—C2	125.90 (19)	C5—C12—H12	120.00
C1—C2—C3	123.13 (18)	C11—C12—H12	120.00
C2—C3—C9	118.60 (16)	C10—C13—H131	110.00
C2—C3—C21	118.10 (17)	C10—C13—H132	110.00
C9—C3—C21	123.24 (15)	C10—C13—H133	109.00
O3—C4—C5	120.05 (15)	H131—C13—H132	109.00
O3—C4—C9	117.64 (15)	H131—C13—H133	110.00
C5—C4—C9	122.22 (15)	H132—C13—H133	109.00
C4—C5—C6	118.71 (16)	C10—C14—H141	109.00
C4—C5—C12	117.92 (16)	C10—C14—H142	110.00
C6—C5—C12	123.27 (17)	C10—C14—H143	109.00
O4—C6—C5	119.82 (17)	H141—C14—H142	109.00
O4—C6—C7	119.10 (16)	H141—C14—H143	109.00
C5—C6—C7	120.92 (16)	H142—C14—H143	110.00
C6—C7—C8	118.42 (16)	O4—C15—H151	109.00
C6—C7—C16	120.80 (16)	O4—C15—H152	111.00
C8—C7—C16	120.72 (15)	O4—C15—H153	109.00
O1—C8—C7	115.05 (15)	H151—C15—H152	109.00
O1—C8—C9	120.94 (15)	H151—C15—H153	110.00
C7—C8—C9	124.01 (15)	H152—C15—H153	109.00
C3—C9—C4	126.22 (15)	C17—C18—H18	117.00
C3—C9—C8	118.14 (15)	C19—C18—H18	117.00
C4—C9—C8	115.64 (15)	C18—C19—H191	110.00
O3—C10—C11	109.14 (16)	C18—C19—H192	110.00
O3—C10—C13	107.16 (15)	C18—C19—H193	109.00
O3—C10—C14	103.60 (16)	H191—C19—H192	110.00
C11—C10—C13	111.86 (19)	H191—C19—H193	109.00
C11—C10—C14	112.31 (16)	H192—C19—H193	109.00
C13—C10—C14	112.28 (18)	C17—C20—H201	110.00
C10—C11—C12	121.01 (18)	C17—C20—H202	109.00
C5—C12—C11	119.6 (2)	C17—C20—H203	109.00
O5—C16—C7	118.35 (18)	H201—C20—H202	110.00
O5—C16—C17	121.52 (18)	H201—C20—H203	109.00
C7—C16—C17	120.12 (16)	H202—C20—H203	109.00
C16—C17—C18	119.84 (17)	C21—C22—H22	120.00
C16—C17—C20	116.41 (18)	C23—C22—H22	120.00
C18—C17—C20	123.71 (19)	C22—C23—H23	120.00
C17—C18—C19	126.6 (2)	C24—C23—H23	119.00
C3—C21—C22	120.04 (17)	C23—C24—H24	120.00
C3—C21—C26	121.08 (18)	C25—C24—H24	120.00
C22—C21—C26	118.86 (19)	C24—C25—H25	119.00
C21—C22—C23	120.1 (2)	C26—C25—H25	120.00
C22—C23—C24	120.4 (2)	C21—C26—H26	120.00
C23—C24—C25	119.8 (2)	C25—C26—H26	120.00
C24—C25—C26	120.7 (2)		
			
C8—O1—C1—O2	−177.92 (18)	C6—C5—C12—C11	−168.4 (2)
C8—O1—C1—C2	4.8 (3)	O4—C6—C7—C8	−176.02 (18)
C1—O1—C8—C7	178.65 (17)	O4—C6—C7—C16	6.7 (3)
C1—O1—C8—C9	−1.1 (3)	C5—C6—C7—C8	−0.6 (3)
C10—O3—C4—C5	−28.4 (2)	C5—C6—C7—C16	−177.87 (17)
C10—O3—C4—C9	155.07 (16)	C6—C7—C8—O1	−178.13 (17)
C4—O3—C10—C11	43.8 (2)	C6—C7—C8—C9	1.6 (3)
C4—O3—C10—C13	−77.49 (19)	C16—C7—C8—O1	−0.9 (3)
C4—O3—C10—C14	163.64 (15)	C16—C7—C8—C9	178.89 (18)
C15—O4—C6—C5	79.4 (2)	C6—C7—C16—O5	90.6 (2)
C15—O4—C6—C7	−105.2 (2)	C6—C7—C16—C17	−90.1 (2)
O1—C1—C2—C3	−4.8 (3)	C8—C7—C16—O5	−86.6 (3)
O2—C1—C2—C3	178.1 (2)	C8—C7—C16—C17	92.8 (2)
C1—C2—C3—C9	1.1 (3)	O1—C8—C9—C3	−2.7 (3)
C1—C2—C3—C21	178.37 (18)	O1—C8—C9—C4	176.71 (16)
C2—C3—C9—C4	−176.70 (19)	C7—C8—C9—C3	177.57 (18)
C2—C3—C9—C8	2.6 (3)	C7—C8—C9—C4	−3.0 (3)
C21—C3—C9—C4	6.2 (3)	O3—C10—C11—C12	−31.7 (3)
C21—C3—C9—C8	−174.46 (17)	C13—C10—C11—C12	86.8 (3)
C2—C3—C21—C22	−95.7 (2)	C14—C10—C11—C12	−145.9 (3)
C2—C3—C21—C26	82.5 (3)	C10—C11—C12—C5	3.7 (4)
C9—C3—C21—C22	81.4 (3)	O5—C16—C17—C18	−171.3 (2)
C9—C3—C21—C26	−100.4 (2)	O5—C16—C17—C20	6.4 (3)
O3—C4—C5—C6	−179.18 (17)	C7—C16—C17—C18	9.4 (3)
O3—C4—C5—C12	−2.8 (3)	C7—C16—C17—C20	−172.87 (17)
C9—C4—C5—C6	−2.8 (3)	C16—C17—C18—C19	177.6 (2)
C9—C4—C5—C12	173.62 (19)	C20—C17—C18—C19	0.1 (3)
O3—C4—C9—C3	−0.6 (3)	C3—C21—C22—C23	−178.4 (2)
O3—C4—C9—C8	−179.93 (16)	C26—C21—C22—C23	3.4 (3)
C5—C4—C9—C3	−177.06 (18)	C3—C21—C26—C25	178.2 (2)
C5—C4—C9—C8	3.6 (3)	C22—C21—C26—C25	−3.6 (3)
C4—C5—C6—O4	176.58 (18)	C21—C22—C23—C24	−1.5 (4)
C4—C5—C6—C7	1.2 (3)	C22—C23—C24—C25	−0.3 (4)
C12—C5—C6—O4	0.4 (3)	C23—C24—C25—C26	0.0 (4)
C12—C5—C6—C7	−175.0 (2)	C24—C25—C26—C21	2.0 (4)
C4—C5—C12—C11	15.4 (3)		

### Hydrogen-bond geometry (Å, °)

**Table:**

*D*—H···*A*	*D*—H	H···*A*	*D*···*A*	*D*—H···*A*
C11—H11···O2^i^	0.95	2.44	3.381 (3)	172
C26—H26···O5^ii^	0.95	2.40	3.225 (3)	145

Symmetry codes: (i) *x*, *y*−1, *z*; (ii) −*x*+2, −*y*+2, −*z*+1.

## References

[bb1] Barbour, L. J. (2001). *J. Supramol. Chem.* **1**, 189–191.

[bb2] Betteridge, P. W., Carruthers, J. R., Cooper, R. I., Prout, K. & Watkin, D. J. (2003). *J. Appl. Cryst.* **36**, 1487.

[bb3] Burla, M. C., Caliandro, R., Camalli, M., Carrozzini, B., Cascarano, G. L., De Caro, L., Giacovazzo, C., Polidori, G. & Spagna, R. (2005). *J. Appl. Cryst.* **38**, 381–388.

[bb4] Carruthers, J. R. & Watkin, D. J. (1979). *Acta Cryst.* A**35**, 698–699.

[bb5] Desiraju, G. R. & Steiner, T. (1999). *The Weak Hydrogen Bond in Structural Chemistry and Biology* Oxford University Press.

[bb6] Molecular Structure Corporation & Rigaku (2006). *CrystalStructure* MSC, The Woodlands, Texas, USA, and Rigaku Corporation, Tokyo, Japan.

[bb7] Rigaku (2005). *CrystalClear.* Rigaku Corporation, Tokyo, Japan.

